# Old Dog New Tricks; Revisiting How Stroke Modulates the Systemic Immune Landscape

**DOI:** 10.3389/fneur.2019.00718

**Published:** 2019-07-02

**Authors:** Siddharth Krishnan, Catherine B. Lawrence

**Affiliations:** ^1^Faculty of Biology, Medicine and Health, Manchester Academic Health Science Centre, Lydia Becker Institute of Immunology and Inflammation, University of Manchester, Manchester, United Kingdom; ^2^Manchester Collaborative Centre for Inflammation Research, University of Manchester, Manchester, United Kingdom; ^3^Division of Infection, Immunity and Respiratory Medicine, Faculty of Biology, Medicine and Health, Manchester Academic Health Science Centre, University of Manchester, Manchester, United Kingdom; ^4^Division of Neuroscience and Experimental Psychology, Faculty of Biology, Medicine and Health, Manchester Academic Health Science Centre, University of Manchester, Manchester, United Kingdom

**Keywords:** cerebral ischaemia, post-stroke infection, systemic immunity, innate immune training, neuroimmunology

## Abstract

Infections in the post-acute phase of cerebral ischaemia impede optimal recovery by exacerbating morbidity and mortality. Our review aims to reconcile the increased infection susceptibility of patients post-stroke by consolidating our understanding of compartmentalised alterations to systemic immunity. Mounting evidence has catalogued alterations to numerous immune cell populations but an understanding of the mechanisms of long-range communication between the immune system, nervous system and other organs beyond the involvement of autonomic signalling is lacking. By taking our cues from established and emerging concepts of neuro-immune interactions, immune-mediated inter-organ cross-talk, innate immune training and the role of microbiota-derived signals in central nervous system (CNS) function we will explore mechanisms of how cerebral ischaemia could shape systemic immune function. In this context, we will also discuss a key question: how are immune requirements critical for mediating repair of the ischaemic insult balanced by the need for anti-microbial immunity post-stroke, given that they are mediated by mutually exclusive immune networks? Our reformed understanding of the immune landscape post-stroke and novel mechanisms at play could guide targeted therapeutic interventions and initiate a step-change in the clinical management of these infectious complications post-stroke.

Ischaemic stroke imposes a significant burden on health-care systems and societies across the globe as a consequence of the morbidity and mortality associated with the condition (1, 2). In addition to neurological deficits, infectious complications such as pneumonia and urinary tract infections post-stroke also pose a hurdle to optimal recovery, affecting a significant proportion of patients and exacerbating mortality risk (3–7). Despite significant leaps in our understanding of key players in the immune response post-stroke (8–19) and biomarkers of infection (4, 20, 21), therapy using statins (for immunomodulation), prophylactic beta-blockers (to target sympathetic activation), and antibiotics (to control infection) have proved ineffective in treating infection (22–26). Equally, attempts to improve stroke outcome using neuroprotective therapies to harness the immune system such as dexamethasone, erythropoietin, and many others have been met with limited success in the clinic; in part, due to undesirable effects on systemic immunity. Yet, the question of whether drugs that possess immunomodulatory properties can be purposed for stroke patients remains to be resolved (27). What is clear, however, is the onset of a plethora of immunological alterations involving diverse immune cells and tissues that is essential for both neurological recovery as well as the occurrence of systemic infections through impairments in anti-microbial immunity (13). In this review, we collate previously described alterations to systemic immunity, and delineate how long-range communication between the CNS and the periphery shapes immune control by taking our cues from data that does not examine stroke. We will finally examine whether the cost of tissue repair (of the ischaemic damage) is impairment in anti-microbial immunity and consequently, increased infection susceptibility.

## The Systemic Immune Landscape of Stroke

To date, there is a wealth of information surrounding the spectrum of immune alterations that ensue following a stroke in both patients as well as models of experimental stroke. The onset of immunological alterations in the central nervous system (CNS) is sequential; neutrophils are recruited hours after stroke by activated microglia and endothelial cells, followed by monocytes a few days following the insult, whilst T and B cells infiltrate the ischaemic tissue in the succeeding weeks (28–32). This contrasts immune alterations in peripheral tissues where stroke drives alterations to the hepatic cytokine networks as early as 6 h post-stroke whilst other systemic immune effects present 24 h post-stroke, affecting both myeloid and lymphoid populations (12, 14, 17, 33–35). However, the mechanisms by which these rapidly-elicited immune changes morph into immune suppression remain poorly defined.

As well as directly altering immune networks, stroke-driven systemic cytokine signals have also emerged as key players that have been implicated to have divergent effects on outcome following stroke (16, 36, 37). Importantly, hepatic interleukin (IL)-6 and chemokine (C-X-C motif) ligand 1 (CXCL1) drive a rapid and transient inflammatory response (35, 38) whilst primed splenocytes secrete tumour necrosis factor (TNF)-α, interferon (IFN)-γ, chemokine (C-C motif) ligand 2 (CCL2) and IL-2 (39); and granulocyte colony stimulating factor (G-CSF) mobilises monocytes and neutrophils from the bone marrow following experimental stroke (40, 41). Moreover, stroke has also been shown to compromise humoral responses through the induction of hypogammaglobulinaemia via the excretional loss of immunoglobulin (Ig) G and impairments in innate-like B cell responses facilitating IgM loss (12, 42).

Many groups have implicated the loss of lymphocytes (T and natural killer (NK) cells), termed lymphopenia, in circulation as a central feature of stroke-induced immune suppression in patients (43–45), a hallmark also replicated in experimental stroke (14, 45, 46). It is thought that the remaining T lymphocytes are also fundamentally altered, being primed to mount a type-1 response through increased IFN-γ and IL-2 production even years after the ischaemic insult (47). In fact, experimental models of stroke have also demonstrated that CD4^+^ T cells in Peyer's patches (lymphoid tissue in the small intestine) of mice are primed to secrete increased levels of IL-17 and IFN-γ (15). Similarly, there is also an activation of innate lymphocytes such as invariant natural killer T cells (iNKT) cells in the liver in tandem with a cessation of their patrolling behaviour in the sinusoids (48). Additionally, stroke also leads to the apoptotic loss of splenic marginal zone B cells (12) whilst their loss in the blood and bone marrow is predominantly driven by alterations in lymphopoiesis (49).

As such, even innate immune responses are compromised in stroke patients with impairments in the oxidative burst of neutrophils (34) as well as shifts in the proportions and properties of monocyte populations (9, 17, 50, 51). Specifically, as classical and intermediate monocytes expand within the circulating monocyte pool (50, 51) they accrue deficiencies in their anti-microbial immunity as evidenced by their shedding of CD163, tolerance to endotoxin and their inability to secrete key cytokines such as TNF-α, IL-6, and CCL2. These monocytes, also through their increased secretion of IL-10 and downregulation of human leukocyte antigen-DR isotype (HLA-DR) thus acquire an immune suppressed state (16, 17, 33, 46, 52–54). That said, CD74, the invariant polypeptide chain associated with the HLA complex is upregulated in the peripheral blood mononuclear cells of patients (55). Despite being capable of processing and transporting antigens, monocytes, nevertheless, are not professional antigen-presenting cells (56, 57). Consequently, it remains to be determined if the loss of HLA-DR functionally impacts these monocytes in their ability to prime T cell responses post-stroke. Indeed, murine models of experimental stroke have also implicated impairments in monocyte function with an increase in splenic monocytes; both macrophages and monocytes in the spleen downregulate their expression of major histocompatibility complex II (MHCII) (58, 59). By contrast, the role of non-classical monocytes following stroke is less clear in both patients and experimental models of stroke (60, 61).

## The Dynamic Interactions Between the CNS and Systemic Immunity in Stroke

Thus far, we have discussed compartmentalised alterations to systemic immunity post-stroke. In this section, we shall highlight how recruited immune cells interface with locally resident cells and the milieu of cytokines following ischaemic injury in stroke. The identification of meningeal lymphatics in the dural sinuses of the CNS (62, 63) as well as previously undescribed subsets of CNS-resident immune cells such as type 2 innate lymphoid cells (ILC2) (64) has reformed our understanding of immunity in the CNS and its frontiers. It is particularly salient to probe the role of these ILC2s post-stroke given their established function in mediating type 2 immunity (65–69) and the critical role of type 2 immunity in mediating tissue repair (70–73). Essentially, type 2 immunity is an ancient arm of the immune system that is mobilised during infection by multicellular metazoan parasites such as helminths that can drive tissue injury as they develop in the host (70, 74, 75). Characterised by the cytokines IL-3, IL-4, IL-5, IL-9, IL-10, and IL-13, it can be orchestrated by a large repertoire of cells including expanded populations of macrophages, T cell, ILCs, and other sentinels of tissue injury to mediate tissue repair in parallel with parasite expulsion (73, 76, 77). As a result, it becomes important to appreciate the reciprocal regulation that exists between the CNS and systemic immunity in homeostasis (78–80) and how injury to the brain modulates this cross-talk during stroke.

Local cytokine cues facilitate neutrophil recruitment to the ischaemic hemisphere as early as 6 h post-stroke in an intercellular adhesion molecule-1-dependent manner (28, 81). Neutrophils are thought to exacerbate the initial injury through the release of proteases and neutrophil extracellular traps (NETs) that exert neurotoxic effects (82, 83). In particular, the release of matrix metalloproteinase-9, a gelatinase, is thought to compromise the integrity of the blood-brain barrier (BBB) (84, 85) and promote the development of oedema (86). Despite being short-lived, it is thought that neutrophils can take on polarisation states through the acquisition of Ym1 and CD206 expression which has been suggested to be important for their clearance and the resolution of inflammation post-stroke (87).

The role of microglia through their interaction with peripherally-derived leukocytes adds an extra layer of complexity as they are double-edged swords with the capacity to propagate and curtail inflammation, and support neurogenesis (88, 89). Through the acquisition of a classically activated state as a result of astrocytic signals and/or microglial CD8 signalling, they secrete pro-inflammatory cytokines including TNF-α, IL-1α that induce neurotoxic astrocytes (90) and disrupt the integrity of the BBB post-stroke (91). By contrast, they are also capable of limiting neuroinflammation by restraining neutrophil recruitment through the effects of transforming growth factor (TGF)-β on astrocyte-derived CXCL1 (92), limiting neuronal excitotoxicity (93) as well as myelin auto-reactivity (94).

Being one of the first responders to injury or infection (95–97), it comes as no surprise that monocytes are rapidly recruited from the periphery to the ischaemic brain post-stroke (9, 19, 31, 79). Whilst they can infiltrate the brain parenchyma through a breached BBB (98), studies have also demonstrated that the choroid plexus is another means of access to the CNS (95, 99). By employing the CD73 enzyme for extravasation and transmigration, monocytes are thought to infiltrate the CNS via the choroid plexus through interactions between the vascular cell adhesion molecule 1 (VCAM-1) and very late antigen-4 (VLA-4). In the CNS, monocytes are an acute source of pro-inflammatory cytokines that drive neuroinflammation following which the milieu of cytokines directs their differentiation into macrophages with features of alternative activation such as arginase-1 and Ym1 expression to mediate tissue repair (61, 100–103). Nevertheless, the interactions between microglia and monocyte-derived macrophages are indispensable for restraining and resolution of long-term microglial inflammation (61, 104) and promoting neurological recovery in diverse settings of CNS injury (95, 99). Despite their ability to engraft in this niche and mediate repair in contexts of inflammation, studies have shown that these monocyte-derived macrophages maintain a distinct transcriptional identity and cannot replenish microglia (105–107). Although this has been ascribed to the embryonic origins of microglia that are seeded from the yolk sac (108–110), the dynamics of this niche remain unclear in stroke.

Amongst leukocytes recruited to the infarcted brain, T cells are one of the last responders and their dynamics are equally complex with studies suggesting that their functions *in situ* are influenced by not just the subset but also the route of entry into the CNS (8, 111, 112). It is thought that γδ T cells gain access to the injured brain through the leptomeninges in a C-C chemokine receptor (CCR) type 6-dependent manner (8, 113, 114) meanwhile other T cells can access the CNS through the choroid plexus (112) in addition to migrating across a breached BBB (111). Consequently, γδ T cells exacerbate ischaemic damage through their production of IL-17 (8, 113, 114) whilst CD4^+^ T cells can take on a type 2 activation state and mediate tissue repair through the production of IL-4 in synergy with macrophages (72, 73, 111).

In conjunction with the previously described alterations to systemic immunity it can thus be observed that stroke initiates a complex cycle of events in the ischaemic brain that shapes immunity and inflammation at distal sites. The notion that inflammation at one site can affect other sites is not a new concept as studies have demonstrated that infection-driven inflammation or antigenic challenges in the lungs are capable of promoting the homing of CD4^+^ T cells to the gastrointestinal tract where they can drive pathology or protective immunity (115, 116). In this paradigm, it is worth noting that γδ T cells are recruited from the intestinal tract (8) where stroke also modulates inflammation in the Peyer's patches whilst driving shifts in microbial communities (15). It can therefore be observed that by fuelling neuroinflammation, ischaemic damage concurrently shapes immune networks in peripheral tissues such as the blood, bone marrow, and spleen that in turn amplify neuroinflammation and inter-organ cross-talk.

## The Reaches of Stroke: Means of Immune Control

**Figure 1:**
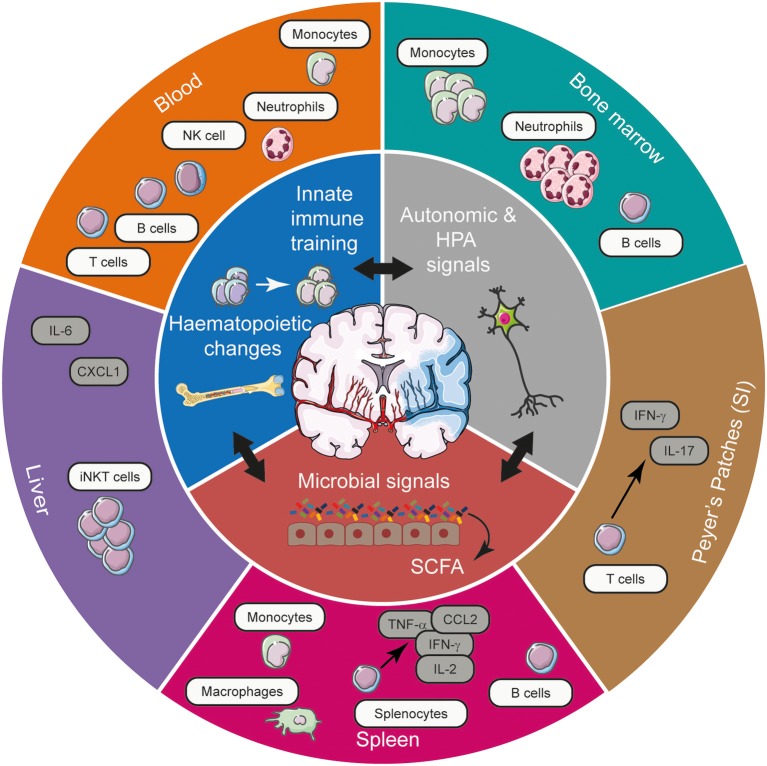
The spectrum of systemic immune alterations post-stroke and the potential mechanisms exploited by stroke to mediate these long-range effects. Stroke, through its effects on autonomic and HPA axis activation, innate immune training, and microbial communities in the gut can gain access to various tissues to shape the systemic immune landscape, affecting cellular, and cytokine networks. Acting in synergy, these means of long-range communication modulate the frequency and functional responsiveness of a plethora of immune cells, tuning the quality of the immune response elicited in various tissues in response to CNS injury. CCL2, chemokine (C-C motif) ligand 2; CXCL1, chemokine (C-X-C motif) ligand 1; IFN-γ, interferon- γ; HPA, hypothalamic-pituitary-adrenal; IL, interleukin; iNKT cell, invariant natural killer T cell; NK cell, natural killer cell; SCFA, short chain fatty acids; SI, small intestine; TNF-α, tumour necrosis factor-α.

Given the diverse range of immune alterations elicited by stroke, it is conceivable that long-range communication mechanisms between the CNS and the peripheral immune system is a prerequisite to mediating these effects. By employing established and emerging immunological data that does not investigate stroke, we will outline plausible mechanisms of how inflammation and injury in the brain in the context of stroke can shape immune networks at distal sites. Thus, we will highlight possible mechanisms that cerebral ischaemia could utilise to effect compartmentalised changes to systemic immunity and add to the concepts of autonomic dysfunction by suggesting avenues of research (Figure 1).

### Autonomic Signalling

Mounting evidence has ascribed the alterations in systemic immunity to be consequence of increased autonomic signalling, particularly an over activation of the sympathetic nervous system (SNS) through the excessive release of catecholamines, in both patients (43, 117, 118) and experimental stroke (12, 14, 48). Studies have also implicated aberrant cholinergic input (45, 119, 120) and glucocorticoid signalling via the hypothalamic-pituitary-adrenal (HPA) axis (14, 49, 121) in mediating systemic immune dysfunction; again through the excessive release of stress mediators such as cortisol and glucocorticoids into the circulation. In addition to being shaped by circulating catecholamines, many tissues that exhibit altered immune profiles post-stroke such as the gut, lung, spleen, and bone marrow are in fact hard-wired to the SNS (39, 122–125). Mechanistically, it has been posited that neurogenic input from autonomic signals as well as the HPA axis could calibrate systemic immunity by the direct effects of stress mediators on immune cells through adrenergic (126, 127), cholinergic (128, 129) and glucocorticoid receptor signalling (130, 131). Intriguingly, the phenomenon of immunosuppression driven by autonomic and HPA axis-derived signals is not restricted to stroke but is also observed in the broader context of CNS injury such as traumatic brain and spinal cord injury (132–134). Hence, a broader understanding of fundamental neuronal pathways linking the CNS and immune system could guide therapeutic interventions to alleviate infectious complications driven by CNS injury.

### Innate Immune Training

Emerging evidence has highlighted the training of innate immune responses by diverse stimuli such as pathogen-associated molecular patterns or cytokines and consequently, the development of immunological memory, a concept traditionally confined to the realms of adaptive immunity (135–144). Given the transient existence of innate cells such as monocytes and neutrophils, it is thought that trained innate immunity is a means to elicit a targeted and enhanced immune reaction to suit host requirements. This has been shown to drive immunometabolic reprogramming via epigenetic rewiring (139, 141) or the replacement of these cells *en masse* through the generation of functionally poised ones via alterations in haematopoietic output (135, 144). To put this in the context of stroke, recent work has demonstrated that microglial training resulting from repeated administration of bacterial endotoxin is capable of curtailing IL-1β secretion as well as microglial activation (and hence neuroinflammation) following cerebral ischaemia (136). Equally, it is plausible that stroke itself could elicit the training of immune cells in the periphery through its effects on the bone marrow. For example, it has been shown that stroke increases the generation of classical monocytes as a consequence of altered myelopoietic output post-stroke (40). As the bone marrow receives rich sympathetic innervation, it can be envisaged that stroke-driven autonomic signals could tune myelopoiesis to facilitate tissue repair by transducing signals through distinct adrenergic receptor subtypes (125, 145–148). Therefore, the predisposition to repair and replace the entire pool of monocytes and the duration of these changes could impact infection susceptibility as well as the response to a subsequent stroke.

### Microbiota-Derived Signals

The interaction of stroke with the commensal microbiota in the gastrointestinal tract is extremely nuanced. Not only does the commensal microbiome play a role in shaping stroke outcome (despite the precise effect being unclear) (8, 15, 18, 149–152) but stroke also mediates shifts in key microbial communities like Firmicutes and Bacteroidetes (153). In homeostasis, the microbiome itself calibrates immunity within the gut microenvironment as well as distal sites through the symbiotic relationship of diverse microbial communities with immune networks by means of microbial metabolites (154–157). In light of data suggesting a detrimental effect of the gut microbiota on stroke outcome (assessed by infarct volume) as well as their role in seeding bacteria that drive pneumonia (8, 149), it therefore becomes difficult to reconcile how commensal bacteria mediate such effects given their indispensable roles in calibrating immunity in homeostasis and injury (158–163). Nevertheless, it is conceivable that the leakiness of the gut barrier induced by stroke (15) could promote the systemic dissemination of pathobionts such as *Enterococcus* spp., *Escherichia coli*, and *Morganella morganii* (149) or result in shifts in key microbial communities that could directly impact systemic immune networks and consequently, stroke outcome (153). Equally, microbial metabolites such as short-chain fatty acids for example, butyrate and propionate are capable of modulating myelopoietic output (increasing the generation of functionally poised monocytes, macrophages and dendritic cells), indicating their ability to train innate immune responses as well (156, 157). In light of these data, it is reasonable to hypothesise that stroke, by shaping gut microbial communities, could also train myeloid responses through the generation of primed progenitors, simultaneously influencing immune networks, shaping both infarct repair and infection susceptibility in distal tissues such as the lungs and bone marrow. However, the precise contributions of stroke in driving shifts in microbial communities and innate immune training remain to be elucidated.

## A Balancing act: Ischaemic Injury Repair vs. Anti-Microbial Immunity

Thus far, we have summarised the plethora of effects that stroke has on systemic immunity as well as the means it employs to shape immune function, taking our cues from established and emerging immunological evidence. Taken together, the net effect of the immune alterations elicited by stroke appear to facilitate the repair of the ischaemic insult given that both mononuclear phagocytes as well as T cells appear to adopt an alternatively activated state (14, 31, 61). This is unsurprising as a large body of evidence has identified critical roles for type 2 immunity in mediating tissue repair, a response conserved across vertebrates (74, 75, 164–166). Studies have shown that type 2 cytokines such as IL-4 and IL-13 promote the alternative activation of macrophages via the expression of molecules such as arginase and Ym1 and facilitate collagen fibril assembly (70, 72, 73, 167). However, mononuclear phagocytes with the predisposition to take on an alternatively activated fate remain in circulation and police the immune responses in various tissues but their plasticity, and hence ability, to classically activate and then mediate antimicrobial immunity is questionable. This is due to the fact that the transcriptional and epigenetic landscapes that drive classical and alternative activation, at least in macrophages, are mutually opposing (168). Although tissue-resident macrophages can reversibly polarise in a GATA6-dependent manner (169), whether these principles apply to monocytes and in the context of stroke requires further investigation.

Viewing the paradigm from the vantage point of an immunologist raises some pertinent questions. Is the cost of repairing the ischaemic damage an impairment of antimicrobial defences? Are the various effects on systemic immunity a means to dampen immune responses to antigens that are now exposed to the immune system through a breached BBB following a stroke? Intriguingly, studies have identified that some contexts of stroke (170–174) and other settings of spinal cord (132) and traumatic brain injury (175–177) could also initiate auto-immunity through antibodies to myelin-derived proteins. As a result, immunosuppression post-stroke could be construed as a means to dampen debilitating auto-immunity post-stroke by skewing immunity towards a type 2 phenotype and limiting the splenic B cell pool to constrain humoral and T cell-mediated immunity towards exposed brain antigens. The simultaneous autonomic activation could educate monocytes to take on features of alternative activation in order to augment repair of the injured brain tissue prior to entry though alterations in haematopoiesis in the bone marrow. An inadvertent consequence of this cascade could be increased infection susceptibility due to inadequate plasticity in the polarisation fate acquired during generation. Thus, the systemic availability of poised mononuclear phagocytes could underpin impaired antimicrobial defences in the lungs but it remains to be determined if this is indeed the case.

## Concluding Remarks

Data from clinical and pre-clinical paradigms of stroke highlight a diverse range of effects on systemic immunity that can be mediated directly and indirectly by the ischaemic damage. An appreciation of not just the changes themselves but the means employed to elicit them could inform the choice of viable therapies to mitigate infections post-stroke by digressing from traditional choices of antibiotics and beta-blockers. It is therefore imperative we re-examine data from clinical trials of immunotherapy in settings of auto-immunity and cancer to appreciate the remit of immunotherapy. By taking cues from these studies, the clinical trials of immunomodulatory therapies in stroke could provide insight into the fundamental mechanisms at play and more importantly, the suitability of immunotherapies for bolstering neuroprotection and antimicrobial immunity in stroke. This in turn could guide approaches for targeted therapies in the clinical management of neuroprotection and infection to improve outcome following stroke.

## Author Contributions

SK conceived the idea for the manuscript. SK and CL wrote and edited the manuscript together.

### Conflict of Interest Statement

The authors declare that the research was conducted in the absence of any commercial or financial relationships that could be construed as a potential conflict of interest.
